# Effects of *Saccharomyces cerevisiae* fermentation products on growth performance, fecal short chain fatty acids, and microbiota of pre-weaning calves

**DOI:** 10.5713/ab.24.0340

**Published:** 2024-10-28

**Authors:** Qian Lei, Zhiqiang Cheng, Maocheng Jiang, Qianbo Ma, Xiaoxiao Gong, Yongjiu Huo, Miao Lin

**Affiliations:** 1 College of Animal Science and Technology, Yangzhou University, Yangzhou, China

**Keywords:** Antioxidant, Fecal Microbiota, Pre-Weaning Calves, *Saccharomyces cerevisiae* Fermentation Products

## Abstract

**Objective:**

This research aims to explore the effects of incorporating *Saccharomyces cerevisiae* fermentation products (SCFP) on growth performance, nutrient digestibility, antioxidant capacity, fecal short-chain fatty acids, and microbial composition of pre-weaning calves.

**Methods:**

Twenty Holstein calves, 10 days old and weighing an average of 48.63±0.91 kg, were randomly assigned to either the control group (CON) or the SCFP group, with 10 calves in each group. The CON group received only a basal diet, while the SCFP group received the starter diet supplemented with 5 g/head/d of SCFP products. The pre-trial period lasted for 5 days, followed by a main experimental period of 45 days.

**Results:**

The SCFP group had significantly higher final weight, average daily gain, and feed efficiency compared to the CON group (p<0.05). Moreover, the SCFP group exhibited increased apparent digestibility of dry matter, crude protein, ether extract, acid detergent fiber, Ca, and P (p<0.05). Additionally, supplementation with SCFP led to elevated content of growth hormone, insulin-like growth factor-1, and glucagon-like peptide-1 in serum. The inclusion of SCFP also raised serum catalase content and reduced serum malondialdehyde content in pre-weaning calves. Furthermore, SCFP supplementation influenced the composition of intestinal microflora by decreasing *Actinobacteriota* abundance and increasing the abundance of *Ruminococcus*, *Lachnospiraceae_AC2044_group*, *Parabacteroides*, and *Butyricimonas*.

**Conclusion:**

The addition of SCFP has a positive impact on the growth performance, nutrient digestibility, antioxidant capacity, and intestinal microflora composition of pre-weaning calves.

## INTRODUCTION

Calves are integral to the agricultural economy, and maintaining their health is crucial for sustainable animal husbandry [1,2]. Throughout calf development, gut microbes play a vital role in the breakdown of feed, absorption of nutrients, and energy generation [3,4]. These microorganisms produce enzymes and other substances that aid in the digestion of nutrients like cellulose, protein, and fat in the feed, ultimately enhancing nutrient absorption and utilization, and thereby supporting calf growth [5,6]. The use of environmentally friendly and safe feed additives in livestock production has gained traction in recent years as a response to the rise of antibiotic resistance due to excessive antibiotic use [7].

*Saccharomyces cerevisiae* fermentation products (SCFP) are produced through the anaerobic fermentation of yeast, resulting in beneficial metabolites such as B vitamins, amino acids, nucleotides, lipids, and organic acids [8]. These metabolites can enhance intestinal metabolism, promote digestive enzyme activity, and improve digestion and absorption. The cell wall of *Saccharomyces cerevisiae* contains active polysaccharides like α-D-glucan and β-D-glucan, which interact with immune cells and bind to bacteria, inhibiting the adhesion and colonization of pathogens in the gastrointestinal tract [9]. For instance, Rients et al [10] reported that SCFP supplementation enhanced the antioxidant capacity and immune system of finishing beef, suggesting a potential link between improved growth rate and changes in antioxidant capacity and immune response. Alugongo et al [11] demonstrated that SCFP supplementation could enhance the intestinal health of pre-weaning calves. Additionally, Centeno-Martinez et al [12] found that supplementation with yeast fermentation products did not significantly alter fecal microbial community structure but tended to increase the uniformity of the microbial community.

Research on the effects of different SCFPs on pre-weaning calves is limited and inconsistent. Furthermore, there is a lack of research on the effects of SCFP supplementation on fecal short-chain fatty acids (SCFAs) in pre-weaning calves. Therefore, this experiment is designed to assess the effects of adding SCFP on the growth performance, nutrient digestibility, serum growth-related hormones, antioxidant indices, and SCFAs of pre-weaning calves. Furthermore, the study incorporates 16S rDNA high-throughput sequencing technology to analyze the impact of SCFP on the fecal microbiota of pre-weaning calves.

## MATERIALS AND METHODS

### Animal, experimental design, diet, and management

All animals were cared for according to protocols approved by Yangzhou University Institutional Animal Care and Use Committee (SYXK (Su) IACUC 2021-0026).

The study was conducted at the Gaoyou Experimental Ranch of Yangzhou University (Gaoyou, China). A total of 20 Holstein calves (10 d after birth, body weight [BW] = 48.63±0.91 kg [mean±standard error of the mean [SEM]) were used. All calves were randomly allocated into 2 treatment groups with 10 calves each and were fed in pens with 2 calves in each pen. The calves in the CON group were only fed a basal diet, while the SCFP group was fed the starter diet supplemented with 5g/head/d SCFP products (NutriTek; Diamond V, Cedar Rapids, IA, USA). The level of SCFP was based on the manufacturer’s recommendation for ruminants.

All the calves were fed thrice daily at 08:00, 14:30, and 21:00. Milk was offered in plastic tubs in equal amounts three times a day, and calves had free access to water. A 5-day adaptation phase was followed by a 45-day experimental period. The whole milk was pasteurized by heating up to 70°C for 30 minutes and then cooled to 39°C to 42°C using cold-water circulation around the container before feeding to the calves according to the pasture feeding system. Weaned from day 4 to day 60. Calves are fed 9 L milk for 4 to 20 days; Feed 10.5 L milk for 20 to 30 days. After 30 days, gradually reduce milk volume to 6 L until the 40th day. Feed 4 L milk twice a day for 40 to 50 days, at 08:00 and 17:00 pm respectively. Feed 3 L milk 10 days before weaning, once a day at 08:00. The starters were fed at 7-d age, and the refusal of the previous day was recorded at 09:00 per day. Oat hay was included in the diet on day 30 of age. The chemical compositions of starters, oat hay, SCFP, and milk are shown in Tables 1, 2.

### Body weight and feed intake

The BW of all calves was measured on the first and final day of the experiment before morning feeding, and the average daily gain (ADG) was calculated according to initial and final BW. The accurate starters and oat hay consumption of each calf were recorded and converted into average daily dry matter intake (ADMI). The feed efficiency (FE) was calculated by dividing ADMI by ADG.

### Blood collection and analyses

Blood samples were collected via jugular venipuncture into 10-mL vacutainer tubes without additive anticoagulant at the end of the experiment before morning feeding. Then, the samples were centrifuged at 1,309.3×g for 15 min, and then serum samples were collected and stored at −80°C until further analysis. The detection of growth hormone (GH), insulin-like growth factor 1 (IGF-1), glucagon-like peptide (GLP)-1, and GLP-2 were determined using ELISA kits (Beijing Huaying Institute of Biotechnology, Beijing, China). Superoxide dismutase (SOD), malondialdehyde (MDA), Total antioxidant capacity (T-AOC), Catalase (CAT), and glutathione peroxidase (GSH-Px) were determined via the colorimetric method according to the performed by a biotechnology testing company (Hengyi Biotechnology Co., Ltd., Jiangsu, China).

### Collection and measurements of fecal samples

Fecal samples were collected aseptically from calves at the end of the experiment. First, sterile gloves are applied, then a sterile spoon is used to remove a fecal sample from the calf’s anus, and finally it is stored in a sterile frozen storage tube. The fecal samples were 10 samples from each group of calves in two groups, and the samples were taken on the last day of the experiment. Diluted sulfuric acid (10%) was mixed with fecal samples (2 mL per 100 g sample) for nitrogen fixation. All feed and fecal samples were oven-dried at 65°C and grounded through a 0.35-mm screen for later determinations. The fecal dry matter (DM), ether extract (EE), crude protein (CP), neutral detergent fiber (NDF), and acid detergent fiber (ADF) were analyzed and determined following the method described by Ji et al [13]. The kit method (China Nanjing Construction Co., Ltd., Nanjing, China) was used to detect calcium and phosphorus concentrations. In addition, acid insoluble ash (AIA) was used as an internal marker to determine the nutrient digestibility, which was calculated using the equation:


$$\text{Nutrient digestibility }(\%)=[1-({\text{A}}_{1}\times {\text{F}}_{2})/({\text{A}}_{2}\times {\text{F}}_{1})]\times 100$$

according to Álvarez-Rodríguez et al [14].

Where A_1_ and A_2_ are the nutrient content in fecal and diet (%), F_1_ and F_2_ are the AIA content in fecal and diet (%), respectively.

### Fecal scoring

During the experiment, as shown in Table 3, according to the fecal scoring standard proposed by Larson et al [15], the daily fecal situation of calves was recorded. The lower the score, the harder the stool and a stool score of 3 and above is recorded as diarrhea. Diarrhea rate (%) =∑ (number of diarrhea × number of diarrhea days)/(total number of diarrheas × number of test days) ×100.

### Fecal short-chain fatty acids determination

First, the fecal samples were thawed, and then 1 g of the sample was diluted with 1 mL of purified water, and centrifuged at 13,400×g for 10 min at 4°C. Then, 0.2 mL of 20% metaphosphate containing 60 mM crotonate was added to 1 mL of the supernatant, mixed, and stored at −20°C overnight. The next day, the samples were centrifuged at 13,400×g for 10min, the supernatant was filtered through a 0.22 μm aqueous phase filter membrane, and finally, 1 μL was injected for analysis. The content of SCFAs was determined by GC9800 basic gas Chromatograph overhead air chromatograph (Shanghai Kechuang Chromatography Instrument Co., Ltd., Shanghai, China). The measurement conditions were as follows: injection port 200°C, column box 80°C, flame ionization detector 220°C, valve heating 60°C, and thermal conductivity detector 110°C.

### Fecal microbiome analysis

On the final day of the experiment, 20 g of rectal feces were taken via the intestinal invasion method 2 h after morning feeding. Next, 2 g of fecal was stored in 5 mL Cryovial Plastic Frozen Test Tubes and snap-frozen in liquid nitrogen for fecal microbiome analysis.

The fresh fecal sample was collected and measured by high-throughput sequencing by Genedenovo Biological Technology Co., Ltd. (Guangzhou, China). Microbial DNA was extracted using the HiPure Soil DNA Kits (or HiPure Stool DNA Kits) (Magen, Guangzhou, China) according to the manufacturer’s protocols. The hypervariable V3–V4 region of the 16S rDNA gene was amplified by polymerase chain reaction (PCR) using the primers 341F: CCTACGGGNGGCWGCAG; 806R: GGACTACHVGGGTATCTAAT, where the barcode was an eight-base sequence unique to each sample. The PCR system contained a 50 μL mixture containing 10 μL of 5×Q5 Reaction Buffer, 10 μL of 5×Q5 High GC Enhancer, 1.5 μL of dNTPs (2.5 mM), 1.5 μL of each primer (10 μM), 0.2 μL of Q5 High-Fidelity DNA Polymerase, and 50 ng of template DNA. Related PCR reagents were from New England Biolabs (Ipswich, MA, USA). PCR conditions included pre-denaturation at 95°C for 5 min, followed by 30 cycles at 95°C for 1 min, 60°C for 1 min, and 72°C for 1 min, and a final extension at 72°C for 7 min. Amplicons were extracted from 2% agarose gels and purified using the AxyPrep DNA Gel Extraction Kit (Axygen Biosciences, Union, CA, USA) according to the manufacturer’s instructions and quantified using ABI StepOnePlus Real-Time PCR System (Life Technologies, Foster, CA, USA). Purified amplicons were pooled in equimolar and paired-end sequenced (PE250) on an Illumina platform according to the standard protocols.

After raw reads were obtained by sequencing, DADA2 software was used for data filtering and quality control. Amplicon sequence variants with single-base precision were clustered, which was equivalent to operational taxonomic unit (OTU) clustered with 100% similarity. Community composition analysis, indicator species analysis (observed OTUs, Rank Abundance curve, and linear discriminant analysis effect size [LEfSe] analysis), Alpha diversity analysis (Observed_species, Chao1, Shannon, Simpson, Pielou’s evenness index), and Beta diversity analysis (principal coordinates analysis [PCoA] analysis and Anosim) were performed using QIIME2 [16].

### Statistical analysis

A completely randomized test design was used in the study. All data were analyzed using the independent sample t-test of the IBM SPSS Statistics software (version 25.0). Data are shown as means and SEM. Differences with p<0.05 and p<0.001 were considered significant and highly significant, respectively. The 16S rDNA sequencing data were analyzed on a free online platform of Omicsmart tools (https://www.omicsmart.com/tools). p<0.05 indicated a statistically significant difference, and p<0.01 indicated a statistically significant difference.

## RESULTS

### Growth performance

As shown in Table 4. The initial BW didn’t show a difference (p>0.05) between the CON and SCFP, but at the end of the trial, there was an increase in the final BW in the SCFP group (p<0.05). The ADMI was similar (p>0.05) between the two groups. The ADG gain in the SCFP group was increased (p<0.05). The ADMI-to-ADG ratio in the two groups has a significant difference (p<0.05). Moreover, compared with the CON group, the addition of SCFP decreased the fecal score of pre-weaning calves (p<0.05), and the diarrhea rate was reduced by 24.15%.

### Apparent nutrient digestibility

As shown in Table 5. Except for NDF, the apparent digestibility of DM, CP, EE, ADF, Ca, and P were 76.40%, 62.65%, 65.53%, 49.71%, 67.29%, and 64.77%, which were significantly higher than CON (p<0.05).

### Serum growth-related hormones and antioxidant index

As shown in Table 6. At the end of the trial, the content of GH, IGF-1, and GLP-1 in serum did increase by adding SCFP (p<0.05). Furthermore, the SCFP group exhibited a slightly higher GLP-2 than the CON group (p = 0.070).

The content of SOD, T-AOC, and GSX-Px had no significant effect on the two groups. However, supplementation with SCFP significantly increased the content of CAT and decreased the MDA concentrations in the serum of pre-weaning calves (p<0.05).

### Fecal short-chain fatty acids

As shown in Table 7, there was no significant difference in fecal acetate, isobutyrate, butyrate, valerate, acetate/propionate, and total volatile fatty acids content between the two groups (p>0.05). However, the SCFP group had a significant increase in propionate content and a significant decrease in isovalerate content (p<0.05), and the isobutyrate content tended to decrease.

### Fecal microbiota community

#### OTU statistical analysis

**Figure 1:**
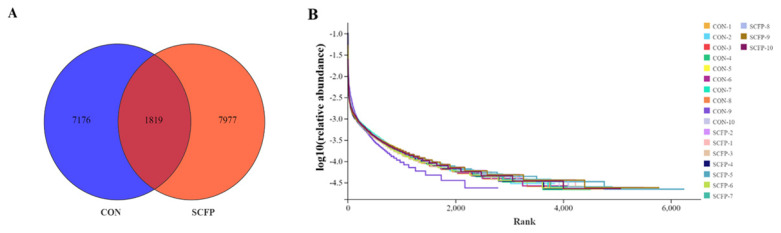
Effects of SCFP on faecal bacterial community in pre- weaning calves. OTU statistical analysis. (A) Venn diagram of OTU in CON and SCFP groups; (B) rank abundance curve (n = 10). CON, control with no SCFP, and fed basal ration. SCFP, fed basal ration, and 5 g/d SCFP (NutriTek; Diamond V, Cedar Rapids, IA, USA) per calf. SCFP, *Saccharomyces cerevisiae* fermentation products; OTU, operational taxonomic unit.

The collected feces were analyzed, as shown in Figure 1, There were 8,995 and 9,796 OTUs in the CON and SCFP groups, respectively. The two groups shared 1,819 OTUs (Figure 1A). The Rank Abundance curve flattened, indicating that the sequencing depth of the sample size was sufficient to cover all species in the sample (Figure 1B).

#### Alpha diversity analysis and beta diversity analysis

**Figure 2:**
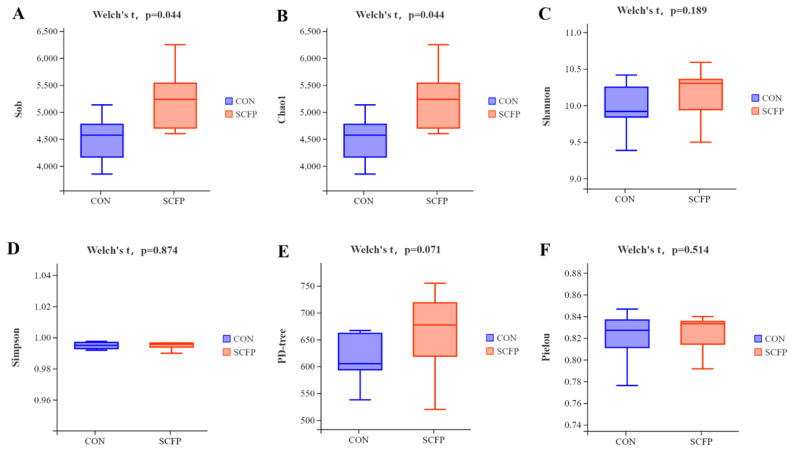
Effects of SCFP on faecal bacterial community in pre-weaning calves. Alpha diversity analysis. (A) Observed_species (Sob) index; (B) Chao1 index; (C) Shannon index; (D) Simpson index. (E) PD whole tree index; (F) Pielou index (n = 10). CON, control with no SCFP, and fed basal ration. SCFP, fed basal ration, and 5 g/d SCFP (NutriTek; Diamond V, Cedar Rapids, IA, USA) per calf. The data were deemed significant if p<0.05 and as tendencies, if 0.05<p<0.10. SCFP, *Saccharomyces cerevisiae* fermentation products.

As shown in Figure 2, the SCFP group had significantly high values in Sob and Chao1 (p<0.05) (Figures 2A, 2B), while Shannon, Simpson, PD-whole tree and Pielou had no significant difference (Figures 2C-2F).

**Figure 3:**
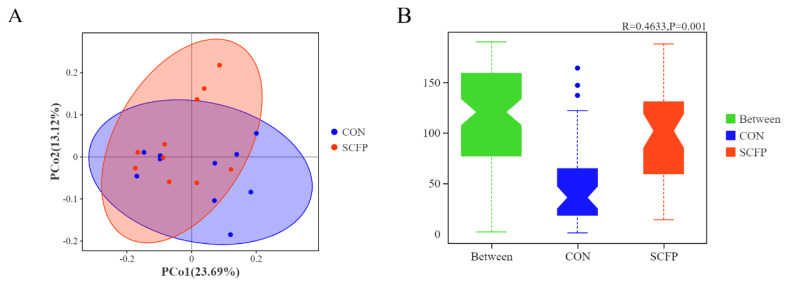
Effects of SCFP on faecal bacterial community in pre-weaning calves. Alpha diversity analysis. Beta diversity analysis. (A) weighted UniFrac-based PCoA plot; (B) unweighted_unifrac-based Anosim plot. (n = 10). CON, control with no SCFP, and fed basal ration. SCFP, fed basal ration, and 5 g/d SCFP (Diamond V, NutriTek, Cedar Rapids, IA, USA) per calf. The data were deemed significant if p<0.05 and as tendencies, if 0.05<p<0.10. SCFP, Saccharomyces cerevisiae fermentation products; PCoA, principal coordinates analysis.

The PCoA at the OTU level of gut microbiota based on weighted_unifrac distance showed the changes in community structure between the two groups. As shown in Figure 3, the contribution values of principal components 1 and 2 were 23.69% and 13.12%, respectively. The results of Anosim based on unweighted_unifrac distance showed that the two groups of samples were grouped reasonably (p<0.05). Therefore, SCFP supplementation had no significant effect on gut microbiota diversity.

#### Differential analysis of fecal microbiota

**Figure 4:**
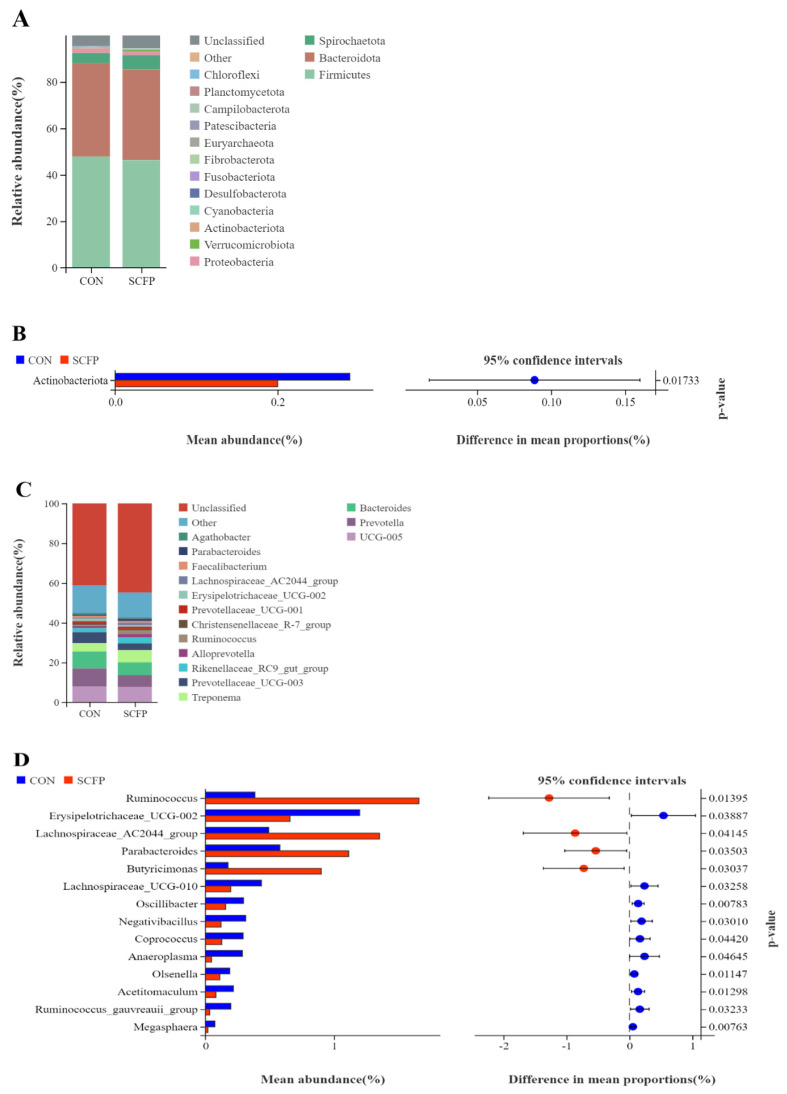
Comparison of the relative abundance of fecal microbiota species distribution for each group in pre-weaning calves (only the top 15 abundant are presented). (A) Phylum level; (B) bar graph of difference by t-test at the phylum level; (C) genus level. Species differences between the two groups were analyzed by Welch’s t-test, and the results were presented as a p-value <0.05 (or 0.01), and a smaller p-value indicated a more significant difference. (D) Bar graph of difference by t-test at the genus level (n = 10). SCFP, *Saccharomyces cerevisiae* fermentation products.

In this study, at the phylum level, the top 10 most abundant species were *Firmicutes*, *Bacteroidota*, *Spirochaetota*, *Proteobacteria*, *Verrucomicrobiota*, *Actinobacteriota*, *Cyanobacteriota*, *Desulfobacterota*, *Fusobacteriota*, and *Fibrobacterota*. The relative abundance of fecal microbiota species distribution for each group is presented in Figure 4A. Additionally, the relative abundance of the *Actinobacteriota* CON group was significantly higher than the SCFP group (p = 0.017) (Figure 4B).

At the genus level, the top 10 most abundant species were *Ruminococcaceae_UCG-005*, *Prevotella*, *Bacteroides*, *Treponema*, *Prevotellaceae_UCG-003*, *Rikenellace-ae_RC9_gut_group*, *Alloprevotella*, and *Ruminococcus*, *Christensenellaceae_R7_group*, and *Prevotellaceae_UCG-001*. The relative abundance of fecal microbiota species distribution for each group is presented in Figure 4C. Additionally, the relative abundance of *Ruminococcus*, *Lachnospiraceae_AC2044_group*, *Parabacteroides*, and *Butyricimonas* in the SCFP group was significantly higher than in the CON group (p<0.05) (Figure 4D).

#### Linear discriminant analysis effect size analysis of fecal microbiota

**Figure 5:**
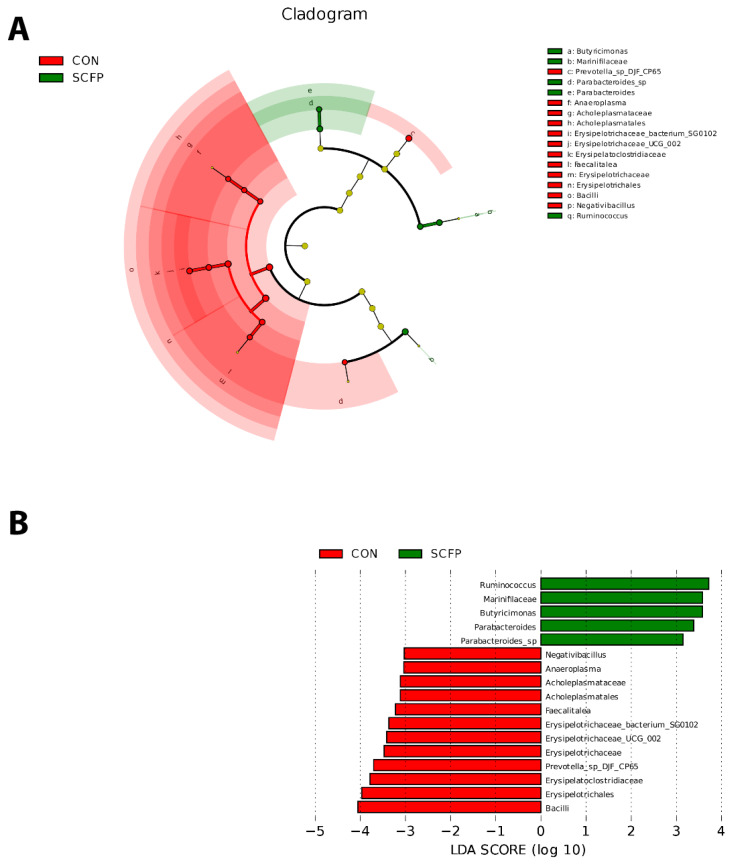
LEfSe analysis was used to further evaluate the effect of SCFP on the composition of the fecal microbiota in pre-weaning calves. (A) Evolutionary branching of the fecal microbiota of pre-weaning calves in CON and SCFP. The red nodes address the microbiome that assumes a significant part in the CON, and the green nodes address the microbiome that assumes a significant part in the SCFP. Species with no tremendous distinction are yellow nodes; (B) correlation of direct discriminant examination (LDA) of the fecal microbiota of pre-weaning calves in CON and SCFP, shows species with contrasts in LDA scores over the set point (3.0), i.e., statistically significant (n = 10). SCFP, *Saccharomyces cerevisiae* fermentation products; LDA, linear discriminant analysis

LEfSe analysis was used to distinguish the modified bacterial aggregates (linear discriminant analysis score > 3.0). In Figure 5A, from outside to inside, each circle successively addresses species at the class, order, family, genus, and species level. Based on this, compared with the CON group, there were three different microbiotas in the SCFP-supplemented group, which were *Butyricimonas*, *Marinifilaceae* (a, b), *Parabacteroides_sp*, *Parabacteroides* (d, e), and *Ruminococcus* (q).

The specific differences between the SCFP and CON groups were as follows: the abundance of *Ruminococcus*, *Marinifilaceae*, *Butyricimonas*, *Parabacteroides*, and *Parabacteroides_sp* in the SCFP group was higher than that in the CON group. On the other hand, in the CON group, the abundance of *Negativibacillus*, *Anaeroplasma*, *Acholeplasmataceae*, *Acholeplasmatales*, *Faecalitalea*, *Erysipelotrichaceae_bacterium_SG0102*, *Erysipelotrichaceae_UCG_002*, *Erysipelotrichaceae*, *Prevotella_sp_DJF_CP65*, *Erysipelatoclostridiaceae*, *Erysipelotrichales*, and *Bacilli* was significantly higher (Figure 5B).

## DISCUSSION

### Growth performance

Numerous studies have demonstrated that feed additives can enhance intestinal microbiota and subsequently stimulate animal growth [17]. This particular study focused on investigating the impact of SCFP on the growth performance of pre-weaning calves, with ADG serving as a key indicator. Takemura et al [18]similarly observed that the addition of *Saccharomyces cerevisiae* led to increased daily weight gain in calves both pre- and post-weaning. The results of this experiment indicated that the SCFP group exhibited significantly higher final weight and ADG compared to the control group. While there was no notable difference in the average daily feed intake of DM between the two groups, the addition of SCFP notably improved the FE. Mitchell and Heinrichs [19]noted that incorporating live yeast culture did not affect feed intake in calves across varying pastures over a period of 7 to 16 weeks, but it did enhance ADG and FE, which can help mitigate the impact of weaning stress on calves. Thus, enhancing the FE is essential for fostering the healthy growth of calves. However, the underdeveloped intestines of calves make them highly susceptible to infections that hinder their growth. Guo et al [20] observed that a blend of Animal Bifidobacterium, Lactobacillus casei, Streptococcus faecalis, and *Saccharomyces cerevisiae* in milk replacer reduces diarrhea in newborn calves. Additionally, the supplementation of SCFP was found to decrease fecal score and diarrhea rate in calves.

### Apparent nutrient digestibility

Nutrient digestibility is a key indicator of calf health and plays a crucial role in promoting their growth. Research by Diao et al [21] revealed that nutrient digestibility in calves tends to increase with age, particularly during the 0 to 2 months period. However, under conditions of weaning stress and dietary changes, the digestibility of DM, CP, and EE may experience a slight decline. Further studies by Zhang et al [22] demonstrated that supplementing with SCFP can enhance the apparent digestibility of DM, CP, NDF, and ADF in calves. Meta-analyses have also shown that SCFP supplementation can lead to an increase in rumen papillae height, ultimately improving nutrient absorption, rumen development, and overall digestibility. Notably, the apparent digestibility of NDF and ADF serves as a reflection of animals’ ability to utilize dietary fiber [23]. In the current study, apart from NDF, the SCFP group exhibited significantly higher apparent digestibility of DM, CP, EE, ADF, calcium (Ca), and phosphorus (P) compared to the control group. These results suggest that the addition of SCFP may enhance animal digestion and nutrient absorption.

### Serum growth-related hormones and antioxidant index

Hormones are essential for regulating growth, energy metabolism, and development. GH plays a role in breaking down bones, organs, muscles, and fat, while also stimulating the synthesis of IGF-1 in most tissues [24]. IGF-1 is crucial for regulating somatic cell growth and development, with its content being influenced by hormone and nutrient content in the body [25]. In a study, the SCFP group showed significantly increased content of serum GH and IGF-1. Additionally, GLP-2 and GLP-1, both derived from proglucagon, impact fat and carbohydrate intake [26]. Burrin et al [27] observed that GLP-1 and GLP-2 content in the SCFP group trended higher compared to the CON group. Overall, it is hypothesized that SCFP may promote serum growth-related hormones and aid in the growth of pre-weaning calves. Furthermore, the body’s antioxidant system is crucial for protecting against oxidative stress damage [28]. T-AOC, SOD, CAT, MDA, and glutathione peroxidase (GSH-Px) are commonly used to assess serum antioxidant capacity [28]. Enzymes like SOD, CAT, and GSH-Px play a key role in reducing free radical formation, while MDA content indicates oxidative stress damage and free radical content in the body [29]. SCFP contains β-glucan and mannosan, possibly through interaction with the protein in the cell wall and other polysaccharide molecules, change the conformation of the molecules or exposed more active site, thus promotes the activity of antioxidant enzymes. In addition, these polysaccharide molecules themselves may also have antioxidant properties capable of directly scavenging free radicals or protecting cells from oxidative damage. The SCFP group exhibited increased serum CAT content and decreased MDA content, suggesting that SCFP can enhance the antioxidant capacity of calves.

### Fecal short-chain fatty acids

SCFAs are important metabolites produced by the gut microbiota, playing various roles in maintaining the health of the host [30]. Acetate, propionate, and butyrate are the most prevalent SCFAs, with acetate primarily produced by *Bacteroidetes* and *Bifidobacteria*, propionate by *Bacteroidetes*, *Firmicutes*, *Bifidobacteria*, and *Salmonella*, and butyrate by *Firmicutes* [31]. An increase in propionate content is associated with improved FE [32]. Isobutyrate and isovalerate have been linked to changes in intestinal cellular morphology and negative effects on intestinal health [33]. Min et al [34] conducted a study on the use of probiotics for treating type 2 diabetes, observing significant alterations in propionate and isovalerate content in fecal. Their experiment showed that the addition of SCFP led to a significant increase in propionate and a decrease in isovalerate content, potentially impacting the intestinal fermentation process in pre-weaning calves and promoting intestinal health.

### Fecal microbiota community

The evolution of the host is influenced by intestinal microbiota, impacting the nutrition and health of calves [35]. In this study, the SCFP group showed a slightly higher number of fecal bacteria OTUs compared to the CON group, with significant increases in Sob and Chao1. Beta diversity analysis revealed that adding SCFP led to changes in the composition of fecal bacteria microbiota, significantly enhancing fecal bacteria diversity. Furthermore, the structural composition and diversity of intestinal microbiota are crucial for maintaining the intestinal microecological environment [36]. *Firmicutes*, *Bacteroides*, *Spirochaetota*, *Proteobacteria*, *Verrucomicrobiota*, *Actinobacteriota*, and *Fusobacteriota* are present in feces, with *Firmicutes* and *Bacteroides* accounting for 90% [37]. *Firmicutes* are important for protein utilization and carbohydrate fermentation, aiding in energy absorption from the diet [38]. In this experiment, pre-weaning calves’ feces had relatively high proportions of *Firmicutes*, *Bacteroides*, *Spirochaetota*, and *Proteobacteria*, with significantly lower content of *Actinobacteriota* in the SCFP group compared to the CON group. Shin et al [39]. discovered a correlation between lower content of *Actinobacteriota* and a healthier intestinal tract, as these bacteria are linked to imbalances in the microbiota. *Actinobacteriota* has been shown to have pathogenic effects, with a significant increase in abundance observed in the feces of goats suffering from diarrhea [40]. Consequently, a higher abundance of *Actinobacteria* in the microbiota of calf feces may elevate the risk of pathogen infection. At the genus level, *Lachnospiraceae* has been found to have a negative correlation with intestinal inflammation [41]. *Ruminococcus* is known for its ability to degrade cellulose and starch, and it plays a role in actively regulating intestinal homeostasis and the immune system [42]. *Parabacteroides* has been associated with anti-obesity effects, as well as improvements in hyperglycemia and insulin resistance [43]. Additionally, *Parabacteroides* is a key player in the intestinal gluconeogenesis pathway, contributing to the production of glucose and promoting digestion and absorption [44]. In this study, the relative abundance of *Ruminococcus*, *Lachnospiraceae_AC2044_group*, and *Parabacteroides* increased in the SCFP group, suggesting that the intestinal environment created by SCFP supplementation may be favorable for the survival of these beneficial bacteria. Therefore, it is hypothesized that adding SCFP could potentially enhance energy use efficiency and support intestinal health.

## CONCLUSION

Supplementing starter diet with SCFP increased BW, ADG, apparent digestibility of nutrients, and antioxidant capacity in pre-weaning calves, while also decreasing diarrhea rate and fecal score. Furthermore, SCFP supplementation led to higher concentrations of GH, IGF-1, GLP-1, and GLP-2. Additionally, SCFP was found to reduce the abundance of *Actinobacteriota* and increase the abundance of *Butyricimonas*, *Parabacteroides*, *Lachnospiraceae_AC2044_group*, and *Ruminococcus*. These results suggest that SCFP supplementation plays a role in regulating the relative abundance and composition of gut microbiota, ultimately promoting animal growth.

## Figures and Tables

**Table 1:** Chemical compositions of the calf starters, oat hay, and SCFP (% of DM)

Item	Starter	Oat hay	SCFP^1)^
DM	94.00	89.00	91.60
CP	20.08	8.53	18.73
EE	3.25	2.28	1.25
Ash	9.95	9.30	11.92
NDF	23.07	47.34	31.47
ADF	15.21	32.07	22.20
Ca	1.12	0.47	0.67
P	0.62	0.28	0.76
NaCl	0.60	-	0.15
MOS	-	-	2.00

Values are presented as mean±standard error of the mean (n = 10).

1) NutriTek; Diamond V, Cedar Rapids, IA, USA.

SCFP, Saccharomyces cerevisiae fermentation products; DM, dry matter; CP, crude protein; EE, ether extract; NDF, neutral detergent fiber; ADF, acid detergent fiber; Ca, calcium; P, phosphorus; NaCl, sodium chloride; MOS, mannosan.

**Table 2:** Nutrient level of milk

Compositions	Content
Milk fat (%)	3.91
Milk protein (%)	3.41
Milk lactose (%)	5.46
Total solids (%)	13.60
SCC (×10^3^/mL)	374.00
MUN (mg/dL)	10.70

SCC, somatic cell; MUN, milk urea nitrogen.

**Table 3:** Calf fecal scoring scale

Outline	Mobility	Score
Normal	Firm but not rigid, slightly altered in shape when dropped or deposited	1
Soft	Unable to maintain shape, clumpy but slightly floppy	2
Ointment	Pancake-shaped, easy to spread to 6 mm thick	3
Aqueous	Liquid, like orange juice, fecal water has a separation phenomenon	4

**Table 4:** Effects of SCFP on growth performance in pre-weaning calves^1)^

Item	CON^2)^	SCFP^3)^	SEM	p-value
Initial BW (kg)	51.80	53.20	2.23	0.564
Final BW (kg)	86.05	92.60	3.09	0.048
ADG (g/d)	761.11	875.56	36.96	0.006
ADMI (g/d)	1,305.47	1,327.99	65.69	0.736
FE (g/g)	1.72	1.52	0.09	0.030
Fecal score	1.28	1.18	0.04	0.021
Diarrhea rate (%)	7.33	5.56	–	

1) The growth data have been published in the Chinese Journal of Animal Science [45].

All data were analyzed using the independent sample t-test and listed as means and standard error of the mean (n = 10).

The data were deemed significant if p<0.05 and as tendencies, if 0.05<p<0.10.

2) Control with no SCFP, and fed basal ration.

3) Fed basal ration, and 5 g/d SCFP (NutriTek; Diamond V, Cedar Rapids, IA, USA) per calf.

SCFP, *Saccharomyces cerevisiae* fermentation products; SEM, standard error of the mean; BW, body weight; ADG, average daily gain; ADMI, average daily dry matter intake; FE, feed efficiency.

**Table 5:** Effects of SCFP on apparent nutrient digestibility of pre-weaning calves (%)^1)^

Digestibility	CON^2)^	SCFP^3)^	SEM	p-value
DM	70.77	76.40	0.96	<0.001
CP	59.83	62.65	1.15	0.024
EE	59.37	65.53	2.19	0.011
NDF	50.30	53.62	2.63	0.225
ADF	43.09	49.71	2.88	0.034
Ca	62.08	67.29	1.77	0.009
P	53.69	64.77	4.02	0.013

1) All data were analyzed using the independent sample t-test and listed as means and standard error of the mean (n = 10).

The data were deemed significant if p<0.05 and as tendencies, if 0.05<p<0.10.

2) Control with no SCFP, and fed basal ration.

3) Fed basal ration, and 5 g/d SCFP (NutriTek; Diamond V, Cedar Rapids, IA, USA) per calf.

SCFP, *Saccharomyces cerevisiae* fermentation products; SEM, standard error of the mean; DM, dry matter; CP, crude Protein; EE, ether extract; NDF, neutral detergent fiber; ADF, acid detergent fiber; Ca, calcium; P, phosphorus.

**Table 6:** Effects of SCFP on serum growth-related hormones and antioxidant index of pre-weaning calves^1)^

Item	CON^2)^	SCFP^3)^	SEM	p-value
GH (ng/mL)	11.61	16.45	1.57	0.007
IGF-1 (ng/mL)	210.87	257.12	5.82	<0.001
GLP-1 (pmol/L)	25.07	33.53	1.56	<0.001
GLP-2 (pmol/L)	411.89	487.35	39.01	0.070
SOD (U/mL)	108.04	114.83	4.34	0.136
MDA (μmol/L)	43.62	26.81	2.04	<0.001
T-AOC (mM/L)	3.54	3.48	0.04	0.182
CAT (U/L)	31.80	35.14	0.81	0.001
GSX-Px (U/mL)	151.58	139.42	7.01	0.100

1) All data were analyzed using the independent sample t-test and listed as means and standard error of the mean (n = 10).

The data were deemed significant if p<0.05 and as tendencies, if 0.05<p<0.10.

2) Control with no SCFP, and fed basal ration.

3) Fed basal ration, and 5 g/d SCFP (NutriTek; Diamond V, Cedar Rapids, IA, USA) per calf.

SCFP, *Saccharomyces cerevisiae* fermentation products; SEM, standard error of the mean; GH, growth hormone; IGF-1, insulin-like growth factor 1; GLP-1, glucagon-like peptide-1; GLP-2, glucagon-like peptide-2; SOD, superoxide dismutase; MDA, malondialdehyde; T-AOC, total antioxidant capacity; CAT, catalase; GSH-Px, glutathione peroxidase.

**Table 7:** Effects of SCFP on fecal short-chain fatty acids of pre-weaning calves^1)^

Item	CON^2)^	SCFP^3)^	SEM	p-value
Acetate (mmol/L)	31.25	33.59	1.62	0.167
Propionate (mmol/L)	8.22	9.51	0.61	0.047
Isobutyrate (mmol/L)	0.88	0.47	0.20	0.054
Butyrate (mmol/L)	3.38	3.93	0.58	0.357
Isovalerate (mmol/L)	0.46	0.23	0.08	0.007
Valerate (mmol/L)	0.55	0.46	0.18	0.629
Acetate/Propionate	3.89	3.57	0.26	0.247
TVFA (mmol/L)	44.96	48.39	2.21	0.137

1) All data were analyzed using the independent sample t-test and listed as means and standard error of the mean (n = 10).

The data were deemed significant if p<0.05 and as tendencies, if 0.05<p<0.10.

2) CON, control with no SCFP, and fed basal ration.

3) SCFP, fed basal ration, and 5 g/d SCFP (NutriTek; Diamond V, Cedar Rapids, IA, USA) per calf.

SCFP, *Saccharomyces cerevisiae* fermentation products; SEM, standard error of the mean; TVFA, total volatile fatty acids.
